# 火场助燃剂检验鉴定的干扰研究进展

**DOI:** 10.3724/SP.J.1123.2021.10003

**Published:** 2022-05-08

**Authors:** Guo YIN, Peiwen QIAN, Fanzi LIQIU, Jing JIN, Ling LIU, Jinzhuan ZHANG

**Affiliations:** 1.中国人民警察大学研究生院, 河北 廊坊 065000; 1. The Graduate School, China People's Police University, Langfang 065000, China; 2.中国人民警察大学侦查学院, 河北 廊坊 065000; 2. School of Criminal Investigation, China People's Police University, Langfang 065000, China

**Keywords:** 火灾调查, 助燃剂, 鉴定, 干扰, 综述, fire investigation, accelerant, identification, interference, review

## Abstract

火灾是影响公共安全最为常见的灾害之一,而放火更是严重威胁人民群众生命财产安全,属于典型的暴力犯罪。犯罪嫌疑人为了达到有效快速放火的目的,往往使用助燃剂实施放火,因而助燃剂的检验鉴定对于认定火灾性质起着至关重要的作用。然而火场情况复杂,容易对助燃剂物证检验鉴定产生较大干扰。在火灾发生发展的过程中,火场高温热环境会作用于已形成的助燃剂燃烧残留物,造成助燃剂的特征组分发生不同程度地挥发、热解等变化,从而影响其检出;同时火灾现场存在的大量石油化工产品,其燃烧/热解产物与常见的助燃剂存在相似甚至相同的特征组分,对判断现场是否存在助燃剂有着很大干扰;而在火灾扑灭之后,助燃剂燃烧残留物在火灾现场还会受到常温环境中光照、压力、通风等因素的共同作用发生物理挥发,其特征组分的质量分数会随之发生下降从而对助燃剂鉴定产生影响;特别地,土壤类物证因土壤中有着不计其数的微生物,它们会使存在于土壤中的助燃剂特征组分发生降解,导致助燃剂组分的减少或缺失,严重影响助燃剂鉴定的准确性。该文从火场热环境、基质干扰、风化效应以及微生物效应4个方面梳理了火场条件对助燃剂检验鉴定干扰的研究现状,重点介绍了火场热环境、橡胶及其制品作为典型基质对汽油检验鉴定影响的新成果,同时指出现阶段此领域研究的不足,并对研究方向进行了展望,旨在为火场助燃剂物证检验鉴定提供参考。

## Abstract (en)

Fire is one of the most common disasters threatening public safety and social development; arson, a typical violent crime, is a serious threat to people's lives and property. Suspects are likely to use accelerants to commit effective and rapid arson. Therefore, the identification of accelerants is of crucial importance in determining the nature of a fire. Fires are commonly complex and intricate, leading to remarkable interference in accelerant identification. During the development of a fire, thermal environment influences the preformed accelerant combustion residues, causing the characteristic components of the accelerant to volatilize/pyrolyze to different degrees, thus interfering with accelerant identification. There are numerous petrochemicals with characteristic components similar to those of accelerants in fire scenes, also contributing to the interference in the determination of the presence of accelerants. After the fire is extinguished, the combustion residues of the accelerant in the fire scene are influenced by the combined effects of heat, light, and pressure in the ambient environment, resulting in the loss of the mass fraction of characteristic components, mainly in the form of volatilization. In particular, with countless microorganisms in the soil, the characteristic components of the present accelerants are degraded, resulting in the reduction or absence of some components, which seriously influences the accuracy of accelerant identification. This study describes the progress of research on the interference in accelerant identification induced by various factors at fire scenes from four aspects: thermal environment, matrix interference, weathering effect, and microbial degradation, with emphasis on the interference from the first aspect. Based on the correlation between the matrix chemical composition/structure and interference degree, new results pertaining to the interference source in accelerant identification are also considered. The shortcomings of current research and the prospects for future research are put forward to provide a reference for accelerant identification at fire scenes. Many studies have been conducted on the above four types of interference in recent years, which provide important reference for accelerant identification regarding the arson caused by ignitable liquids. However, with the application of new materials and techniques, fire scenes have become increasingly complex, which demands higher requirements for accelerant identification in forensic science. Research on thermal environment interference is still in its infancy, and it is of great practical significance to conduct this research on other types of accelerants coupled with other interference factors. In addition, the principle of most of the current research on selecting objects is based on common appearance at fire scenes, rather than the functional group of the chemical structure of the matrix. Systematic and in-depth research on the interference law and mechanism is urgently required, and it is vital to further explore the interference law of the fire background from the perspective of the chemical composition and chemical structure of the substrate. Compared to foreign countries, there is less research on the interference of weathering and microbial effects on ignitable liquid identification in China. Most importantly, there is a lack of unified provisions, such as standard experimental methods to carry out such research, making it urgent to establish relevant standards and specifications. Additionally, while weathering is caused by multiple factors, the existing research on the interference of the weathering effect with ignitable liquid identification focuses merely on volatilization. Therefore, a more accurate experimental design is necessary. Improving the database of interferents under fire conditions, and combining the application of the chemometrics method for the artificial intelligence identification of the combustion residue spectrum, is an effective way to further improve the identification efficiency and accuracy.

火灾是发生概率较高的灾害之一,严重威胁公共安全,而放火作为所有八大暴力犯罪之一,更是对人们生命财产安全和社会稳定造成极大危害,所以快速准确地侦破放火案件,是公安机关和消防救援机构的一项非常重要的任务。在放火案件中,嫌疑人通常会使用汽油等助燃剂来达到快速放火的目的。因此,火场助燃剂的检验鉴定便成为侦破案件的关键,但由于实际火场的复杂性,许多因素会影响助燃剂的鉴定,从而影响司法鉴定结果的准确性。

在火灾发生发展和蔓延阶段,助燃剂燃烧残留物不仅受到基质载体燃烧/热解产物的干扰^[1⇓⇓⇓⇓⇓-7]^,而且也不可避免地受到火场高温环境热量的影响^[8⇓-10]^;灭火后,火场常温环境中的风化效应^[11⇓⇓⇓-15]^和土壤介质的微生物效应^[16⇓⇓-19]^又会进一步影响助燃剂燃烧残留物的特征成分,其中基质载体燃烧/热解产物的影响、火场高温环境热量的影响以及风化效应在每个火场中均存在,而微生物效应仅存在于土壤类物证中^[20⇓⇓-23]^。另外,在技术方法方面,色谱一直是助燃剂检验鉴定领域应用最广泛的分析方法。虽然近些年发展起以色谱为基础的多种联用技术,但无论是国内还是国外标准,都仍将气相色谱-质谱联用(GC-MS)作为检验鉴定助燃剂最为常用和有效的方法。近几年国内外学者应用色谱技术围绕火场背景对助燃剂物证检验鉴定的干扰展开了系列研究,特别是从能量干扰的角度系统提出火场热环境对助燃剂物证存在干扰的相关研究,进一步拓展了火场背景干扰的范围。因此,笔者从火场热环境、基质干扰、风化效应以及微生物效应4个方面对火场中助燃剂检验鉴定干扰的最新研究进行梳理,指出现有研究的不足,并对下一步研究方向进行了展望,以期为火灾调查人员出具鉴定意见提供指导和帮助,推动火场物证检验鉴定研究的发展。

## 1 火场热环境干扰

文献^[8⇓-10]^系统报道了火场热环境对汽油燃烧残留物特征组分的影响。对于使用了助燃剂(如汽油)实施的放火火灾现场,其起火过程一般是助燃剂最先被点燃,先形成一定量的助燃剂燃烧残留物,在助燃剂燃烧过程中产生的能量会引起基质载体或周围可燃物发生热解并燃烧,而这些燃烧的基质载体和周围可燃物释放的热量又会通过辐射或对流的方式对之前形成的助燃剂燃烧残留物物证产生再加热作用,从而对助燃剂特征组分造成一定的干扰,此过程即为火场热环境对助燃剂物证的干扰性。这一干扰作用主要发生在火灾发生的初始阶段,是距离助燃剂泼洒位置较近的可燃物燃烧时产生的热量对助燃剂燃烧残留物的影响,其本质是助燃剂燃烧残留物特征组分的热稳定性。

随着受热程度的增大,汽油燃烧残留物的特征组分可能会随之发生蒸发、热解等物理与化学的变化^[8]^。Jin等^[9]^对92^#^汽油燃烧残留物进行不同程度的再加热,发现火场中不同温度对汽油燃烧残留物各种特征组分的影响程度是不同的。随着汽油燃烧残留物受热程度的增加,燃烧残留物的总离子流图(TIC)中特征峰面积和峰高度均降低(见图1),峰数减少,意味着随温度的升高,残留物特征组分含量减少,种类缺失;其中,汽油特征组分中多环芳烃和茚满更容易受到热破坏,而烷基苯及稠环芳烃等相对稳定,但在600 ℃再受热2 min后,汽油燃烧残留物的所有目标化合物几乎都不能被有效检出^[8,9]^。另外,乙醇汽油在不同二次加热温度下同样呈现出总体特征组分含量减少的趋势,其中烷基苯相对稳定^[10]^。

**图1:**
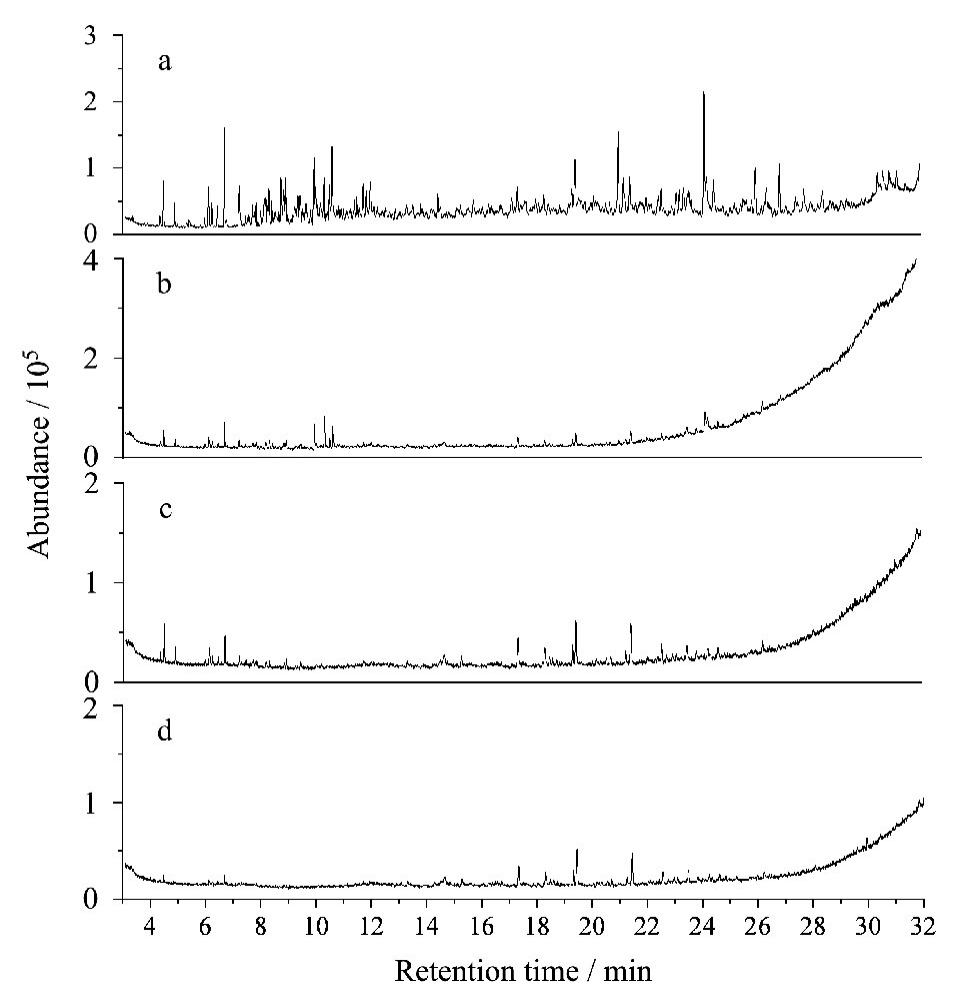
汽油燃烧残留物未再受热及在不同温度下二次受热2 min后的总离子流图^[9]^

火场热环境干扰发生在助燃剂放火火灾的发生发展蔓延阶段,因而这种干扰作用对每个助燃剂放火现场助燃剂燃烧残留物物证而言都是不可避免的,需要特别注意的是,处于高位的物证更容易受到火场热环境干扰。在火场汽油燃烧残留物物证检验鉴定过程中,应当充分考虑到热环境对其产生的干扰,从而更加科学地分析鉴定结果以提高鉴定意见的准确性。

## 2 基质干扰

塑料、纤维、橡胶等典型的高分子有机物是火场常见的可燃物,与助燃剂同属于石油化工产品,这些材料在火灾高温作用下的热解/燃烧产物可能会对助燃剂的检验鉴定产生干扰,这种效应称为基质干扰^[1⇓⇓⇓⇓⇓-7]^。国内外学者针对基质干扰开展了广泛的研究,下面围绕塑料、纤维和橡胶3类典型高分子材料对助燃剂鉴定的干扰进行介绍。

### 2.1 塑料干扰

关于塑料对助燃剂燃烧残留物鉴定的干扰报道较多,但研究对象主要集中在聚乙烯、聚丙烯、聚苯乙烯、聚氯乙烯、乙烯-醋酸乙烯共聚物等常见塑料制品上^[23,24]^。聚乙烯、乙烯-醋酸乙烯共聚物的热解产物中含有C8~C20的正构烷烃,与柴油特征组分较为一致;聚丙烯的热解和燃烧过程比聚乙烯更复杂,其热解主要产物为较低碳数的不饱和支链烯烃(C8~C12);聚苯乙烯热解生成的甲基苯、对二甲苯以及乙基苯均为汽油的特征化合物,对汽油的辨识产生一定干扰;聚氯乙烯燃烧产物为苯、甲基苯、乙基苯、邻二甲苯、1-乙基-2-甲基苯、萘、2-甲基萘等^[25,26]^。由此可见,火场中常见的塑料制品的部分燃烧产物与汽油等助燃剂特征组分相同,对其检出存在一定的影响,但按照相关标准规定^[27,28]^,燃烧残留物中检出助燃剂的部分特征组分并不足以认定存在助燃剂,以汽油为例,我国国标中明确指出其燃烧残留物中主要含烷烃、烯烃、烷基苯、茚满类、稠环芳烃以及多环芳烃等多类化合物,而大部分塑料制品燃烧残留物中主要检测出C10~C20的正构烷烃和烯烃,所以其对汽油鉴定的干扰程度有限,仍可以通过定性分析对汽油等助燃剂和常见塑料进行有效区分^[25,29]^。

### 2.2 纤维干扰

由石油化工原料合成的化纤地毯受热后会热解产生挥发性有机化合物。事实上,日常生活中使用的大部分地毯燃烧/热解产物中经常会检测出甲基苯、乙基苯、1,3-二甲苯等组分,表明地毯对汽油的检出存在一定的干扰^[30,31]^。除了对汽油鉴定存在干扰,棉布、麻纱等天然纤维材料与柴油混合燃烧后,会生成一些更高碳数的烷烃,且烯烃含量较柴油燃烧残留物有所增加,对柴油的检出存在一定的影响,但因为柴油燃烧残留物中正构烷烃、环烷烃、异构烷烃以及芳香烃等成分的特征峰保留时间分布较为规律且稳定,所以仍然可以通过柴油极具特征的谱图对其进行准确认定^[32]^。

由于基质材料的性质各异,其对助燃剂的浸润性不同,Cavanagh等^[31]^对机动车地毯中残留汽油的稳定性情况进行了研究,结果表明少量的(<100 μL)汽油在24 h后已无法被有效检出;燃烧发生后,助燃剂燃烧残留物特征组分在不同基质材料上的存留时间也会有所差异,Dhabbah等^[33]^对汽油和柴油加载在几种纤维材料上形成的燃烧残留物的有效检出时间进行了对比研究:通过将2 mL汽油(或柴油)与多种厚度(5 mm)相同的地毯混合燃烧,待其自然熄灭后,对不同地毯燃烧残留物特征组分的保留情况进行了分析比较,研究发现汽油和柴油加载在羊毛和丝绸上燃烧后的有效存留时间比在棉、涤上的更长^[34]^;但该团队最近研究发现化纤尼龙和合成聚酯纤维与汽油混合燃烧后,残留物的有效检出时间比天然材料(棉花和羊毛)更长,造成这一差异的原因可能是实验条件与变量控制有所不同,另外可能与纤维材料燃烧后形成的残留物对汽油残留物成分的吸附能力强弱不同有关^[35]^。

### 2.3 橡胶干扰

送检的火场燃烧残留物物证中很多是橡胶相关的燃烧残留物,为了在火灾物证鉴定过程中排除橡胶对助燃剂检验鉴定的干扰,许多专家学者选择不同种类橡胶制品开展研究。特别是在车辆火灾中,无论是否发生燃油泄漏(火势较为猛烈或者交通事故机械碰撞等较为特殊的情况会导致油箱受损出现燃油泄漏参与燃烧的情况,但在绝大多数的车辆火灾中不会发生油箱油品泄漏),车辆内外的燃烧残留物都是重要物证,而汽车轮胎燃烧残留物是车辆火灾中提取送检的主要物证之一。轮胎中橡胶成分占比高达93%以上^[36]^:外胎通常为天然橡胶、丁苯橡胶(SBR)和异戊橡胶等,内胎为丁基胶和乙丙橡胶等。余志超^[37]^对4种不同品牌的汽车轮胎(韩泰K415、佳通Comfort T20、固特异65Y系列以及锦湖TX61)展开研究,并结合ASTM E1618-19^[28]^中规定的15种汽油特征组分分析了其对汽油鉴定的干扰,结果表明轮胎燃烧产物中均检出烷基苯、稠环芳烃类特征组分,对分析判断燃烧前轮胎上是否存在汽油具有较大干扰,对苯二胺作为橡胶中常见的一种防老剂,是橡胶燃烧残留物中的特征组分;在轮胎与汽油混合自由燃烧的残留物中,用于鉴定汽油关键组分的相对含量与汽油燃烧残留物中对应组分的相对含量相比相差较大,从而对汽油的检出造成较大影响,容易造成假阴性的误判。在另一项关于汽车轮胎对汽油鉴定干扰的研究中,Li等^[38]^围绕汽车放火的典型场景,基于基质化学成分与其燃烧产物相关联的研究出发点对同一轮胎胎面和胎侧的燃烧残留物对汽油鉴定的干扰进行了研究,发现相较于胎侧,胎面燃烧残留物中检出了更多的C4烷基苯,对汽油鉴定的干扰更大。

另外,王健等^[39]^以某商业化丁苯橡胶垫为研究对象发现SBR橡胶垫燃烧残留物中存在多种用于汽油鉴定的特征组分,但是SBR橡胶垫与其加载汽油混合燃烧残留物的特征组分基本相同,只是当存在汽油时,稠环芳烃和多环芳烃含量较少;而针对粉末状原料级SBR1502燃烧残留物的研究指出SBR燃烧残留物中无法有效检出多环芳烃类组分,可依据此特征对SBR和汽油进行区分^[40]^。

基于燃烧化学的原理,化学结构是影响材料燃烧/热解产物的主要因素。根据GB/T 18294.5-2010^[27]^及ASTM E1618-19^[28]^,汽油的特征组分中绝大多数为芳香烃,而芳香烃的官能团为苯环结构,Jin等^[41]^以此为依据指出:本身含“苯环”的基质更容易对汽油检验鉴定造成干扰,通过选择含“苯”基质SBR原料及制品进行系列研究,发现SBR对汽油鉴定的确存在较强干扰;为了进一步确认产生干扰的官能团,又对苯乙烯含量不同的SBR、聚苯乙烯、聚丁二烯以及化学结构不同于SBR的苯乙烯-丁二烯-苯乙烯嵌段共聚物(SBS)对汽油燃烧残留物的干扰性展开研究,研究结果表明不同苯乙烯含量对燃烧产物的影响不同,相同苯乙烯含量而不同化学结构的SBR与SBS色谱图也存在差异。另外,二烯烃结构极不稳定,在燃烧反应中极易相互反应并环化,生成与汽油燃烧残留物相同的特征组分。因此在具有较强干扰性的基质存在的情况下,过分依赖汽油目标化合物进行定性分析容易对汽油检验鉴定产生“假阳性”的错误结论,另一方面,当这些基质与汽油混合燃烧时,产生的混合燃烧残留物色谱图与汽油色谱图相比会发生明显变形扭曲,如图2所示,汽油加载在基质上混合燃烧后的残留物中烷基苯特征组分的相对含量(峰面积相对比值)与单纯的汽油燃烧残留物对应相比均存在较大差异,这一结果对分析燃烧残留物中是否存在汽油具有较大干扰,很可能得出“假阴性”的错误结论^[8,41⇓-43]^。

**图2:**
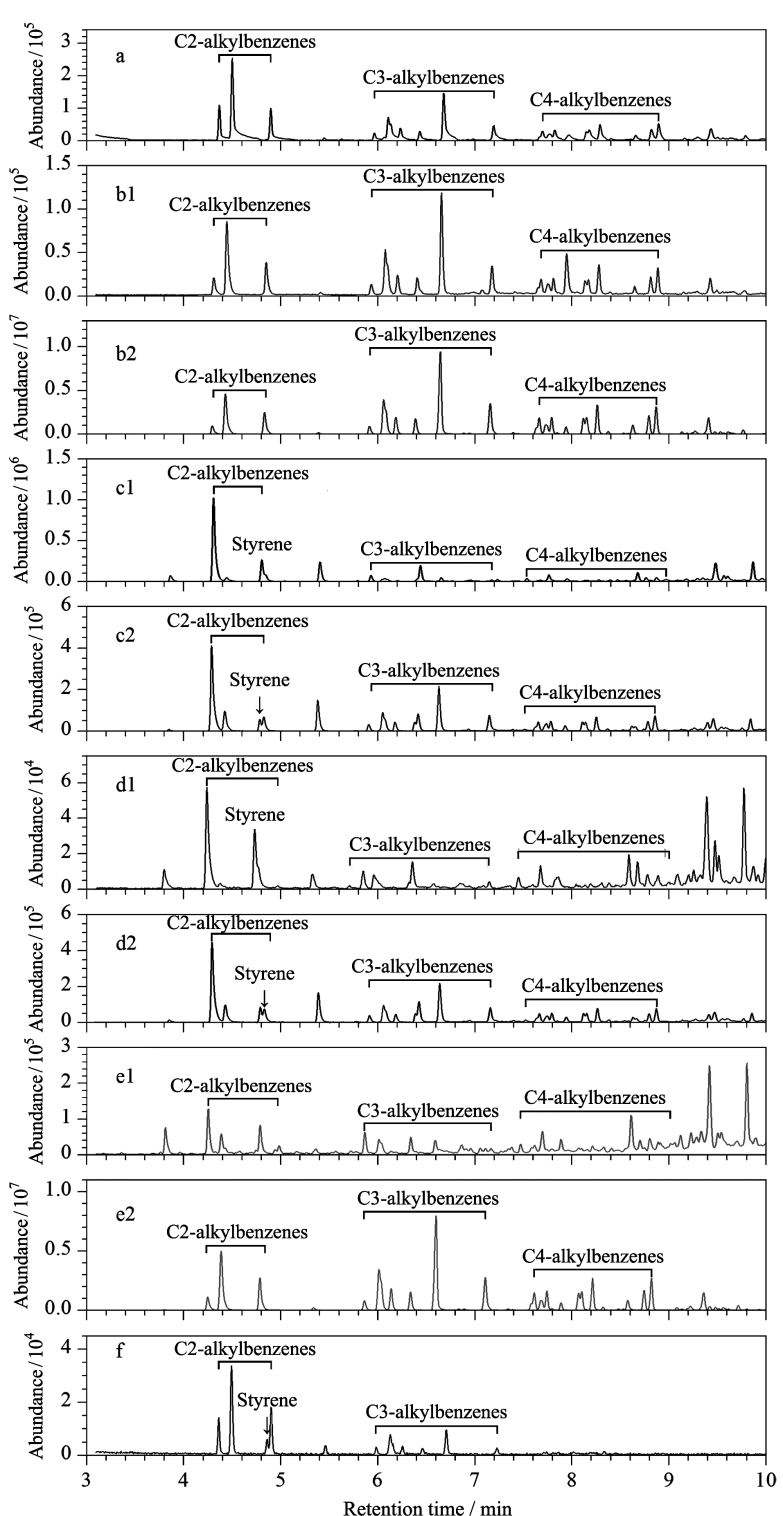
汽油燃烧残留物、基质燃烧残留物以及基质中加载汽油后的混合燃烧样本中烷基苯的提取离子流色谱图^[43]^

## 3 风化效应

火场助燃剂物证的风化效应是指助燃剂物证受到热量、光照、压力等作用而发生的损失,这一过程往往会使助燃剂特征组分产生变化,从而影响助燃剂的检出^[44]^。目前国内外涉及风化效应的研究多以挥发程度^[11,26]^和风化时长^[12⇓⇓-15]^为变量研究风化效应对助燃剂鉴定的影响。

### 3.1 风化效应对助燃剂原样的干扰

虽然助燃剂以原样形式存留在火灾现场的情况并不常见,但原样的研究是助燃剂检验鉴定的基础性工作,对后续开展的一系列鉴定工作起着重要作用。胡何昕等^[11]^采用GC-MS研究了不同挥发程度的92^#^汽油特征组分的变化情况,以质量百分比表示挥发程度,发现当质量损失达75%以后,汽油中绝大部分组分将无法被有效检出,但1,3,5-三甲基苯和乙基苯较为稳定。另外,汽油中不同组分的挥发性按由弱到强依次是:C5烷基苯<萘及其取代物<茚满<C4烷基苯<C1、C2和C3烷基苯^[45]^。另外,残留在衣物上的汽油容易挥发,对于可能存在汽油残留物的衣物物证应做到早发现、早提取、早鉴定;柴油挥发性很弱,在渗透性基质上的存留时间甚至可达几年之久,所以当物证中检测出柴油时,应进一步分析其残留的时间,以确认其是否与案件有直接关联^[46]^。

### 3.2 风化效应对助燃剂燃烧残留物的干扰

在实际助燃剂放火火场中,助燃剂物证很难以原样的形式留存下来,而大多数物证是燃烧残留物。为了更好地结合火场实际,研究人员开展了风化效应对助燃剂燃烧残留物干扰的研究。风化效应主要是助燃剂特征组分挥发性强弱的体现,在针对不同易燃液体燃烧残留物风化效应的研究中,均能观察到较轻的烃类组分首先缺失,色谱图分布偏向于重组分。刘烁彤等^[13,14]^使用GC-MS,比较研究了汽油与橡胶混合燃烧后残留物中各特征峰的出峰时间和峰面积,发现随着提取时间的滞后,特征物质的总含量均出现下降,其中烷基苯、稠环芳烃、多环芳烃3类特征组分较为稳定;而风化效应与基质干扰等其他干扰作用的耦合因涉及多个变量而显得非常复杂,Prather等^[47]^以风化与未风化的汽油加载在尼龙基质上燃烧后的残留物特征组分为对象,比较研究了皮尔逊相关系数、聚类层次分析、主成分分析法3种化学计量学方法在排除风化与基质耦合干扰方面的作用,发现主成分分析法因为能够有效消除基质干扰而在数据分析中较为有效。

此外,酸化汽油是近年来新出现的一种助燃剂,由浓硫酸与汽油混合而成,且与普通汽油在成分上存在着差异。在硫酸的酸化作用下,酸化汽油中会生成一种稳定的特征组分——叔丁基化合物,因此仅仅依赖相关标准^[27,28]^中对汽油组分的规定对酸化汽油进行鉴定可能会导致错误结论的产生。通过对不同风化程度的酸化汽油成分进行比较研究,发现强风化的酸化汽油中已经无法检测出烷基苯等关键组分,但仍然可以明显检出叔丁基化合物,因此叔丁基化合物可以作为强风化样品中鉴别酸化汽油的特征组分^[48]^。Goodman等^[49]^使用GC-MS分析了由4种不同的生物柴油所制得的混合型生物柴油燃烧残留物及以植物油为原料制得的生物柴油燃烧残留物组分情况,结果表明经历了风化作用的生物柴油样品中重烃与脂肪酸甲酯的相对含量明显提高,而轻质组分明显下降。

## 4 微生物效应

火场土壤类助燃剂燃烧残留物是火灾物证鉴定的重要检材,而土壤中的微生物对助燃剂成分的降解给鉴定工作带来了一定困难。目前,国内外关于土壤微生物效应对助燃剂鉴定干扰的研究大多以助燃剂原样为研究对象而展开^[16⇓⇓-19]^,同时也对抑制微生物降解的方法进行了探讨^[50,51]^。

### 4.1 微生物效应对助燃剂鉴定的干扰

土壤中的微生物会降解助燃剂的部分特征组分,特别是C9~C16的正构烷烃和单取代苯,其中偶数碳烷烃比奇数碳烷烃降解率高,而异构烷烃几乎不受影响^[52⇓-54]^; Turner团队在对助燃剂数据参考库^[55]^中50种助燃剂进行研究后发现,微生物会倾向性地对某些化合物结构优先降解,如长链正构烷烃和单取代烷基苯(如甲基苯、乙基苯和丙基苯),甚至一些更长的异构烷烃(例如三甲基辛烷)^[56]^。

不同类型土壤对助燃剂检验鉴定的干扰也不同,Turner团队^[57]^的最新研究表明,住宅区土壤微生物降解活性最高,而用于工业生产的棕地土壤中微生物降解活性最低;方强等^[17]^对比研究了普通土和人工专门配制的培养土(含有丰富养料、具有良好排水和透气性能)中微生物降解对助燃剂检验鉴定的影响,结果表明普通土对助燃剂的降解效应大于培养土的降解效应,正构烷烃和单取代芳香烃相较于异构烷烃更易于受到微生物效应的影响,此结论与Turner团队研究结论一致。

而在另一项关于助燃剂组分降解时间的研究中,Kindell等^[19]^用一种含有14种烃类的混合物代表6类不同的化合物(正构烷烃、异构烷烃、环烷烃、芳香烃、多核芳香烃和含氧化合物),将其放置于90 g的盆栽土壤中进行生物降解,并对降解特定时间的样品组分组成情况进行了GC-MS分析,发现含氧化合物、正构烷烃和甲基苯的损失相当大,混合烃中所有组分累积损失的半衰期为3.15 d,最短仅有1.75 d,由此可见物证鉴定分析人员要充分考虑微生物对助燃剂特征组分的降解时间,避免因为超过组分损失的半衰期而得出错误的鉴定结论。

### 4.2 助燃剂残留物中微生物效应的抑制方法

针对如何抑制土壤中微生物对助燃剂组分的降解,国外展开了较多的探讨。Turner等^[54]^探索了利用杀菌剂对土壤中微生物的处理方案,他们研究了以漂白剂和三氯生为基础的溶液在减缓或消除微生物降解助燃剂方面的效用。但此方法会腐蚀用于盛装样品的容器,并可能进一步导致助燃剂特征组分的缺失;由于氧气是微生物代谢速率的控制因素之一^[58]^, Hutches等^[51]^通过阻碍微生物的代谢来减缓其对助燃剂的降解,此团队提出利用吸氧袋去除土壤类助燃剂物证中的氧气来减缓微生物对助燃剂的降解,实验结果表明,使用此方法可以有效抑制微生物对汽油组分的降解,时效性可长达4周。

综上,微生物效应主要造成苯系物的损失,正构烷烃相较于异构烷烃破坏严重,微生物效应对支链化严重的助燃剂干扰较小。另外,为了避免助燃剂组分受到微生物的降解,造成特征组分减少甚至消失而导致无法被检出,室温下保存的样品应在一天之内进行处理;另一方面,实际火场中助燃剂几乎不以原样的形式保留下来,因此需开展更多以助燃剂燃烧残留物为对象的干扰研究。

## 5 结语

火场助燃剂类物证的检验鉴定主要受火场热环境、基质干扰、风化效应以及微生物效应的影响。随着材料科学和新技术工艺的发展,火场环境会更加复杂,而现阶段对火场助燃剂类物证干扰性的研究仍缺乏深度,还需进行多方面的深入研究。首先,火场热环境对助燃剂检验鉴定干扰的相关研究尚处于起步阶段,针对更多种类的助燃剂开展热环境干扰研究具有重要的现实意义,亟须国内外学者的共同努力。其次,虽然围绕火场背景物对助燃剂鉴定的干扰已经开展了大量研究,但目前的研究在选择研究对象时多以“火场常见”为原则,对干扰规律和干扰机理缺乏深入系统的研究,因此进一步从基质化学成分及化学结构的角度开展对基质干扰助燃剂检验鉴定的规律和机理是该领域重要的研究方向。再次,相较于国外,国内在风化效应和微生物效应对助燃剂鉴定产生的干扰方面开展的研究较少,而且这方面的研究均缺乏统一的实验方法,亟须建立相关的标准规范以增加实验结果的适用性和指导性;风化效应是由多因素作用的结果,而现有的针对火场助燃剂鉴定干扰的研究大多只关注助燃剂本身的挥发性,而更少考虑载体对助燃剂特征组分的吸附性及压力等因素对风化效应的影响,应进一步探讨更多因素以及多因素耦合作用所造成的风化效应对助燃剂鉴定的干扰性。事实上,不仅风化效应本身是多因素的耦合作用,其他干扰作用也会对助燃剂残留物物证先后产生叠加性的影响:火场热环境以及基质干扰均发生在火灾发生发展的过程中,其对助燃剂类物证的影响也不可避免地会在灭火后的风化效应干扰中进一步体现,所以开展多种干扰因素共同作用对助燃剂物证的干扰性研究也是重要的研究方向;另一方面因为干扰物种类繁多、成分复杂,同时火场干扰因素众多,所以开展大量基础研究工作以进一步完善火场助燃剂物证的干扰数据库仍需要国内外学者的共同努力。最后,在色谱技术不断发展的基础上,将更为先进的分析手段借鉴应用到助燃剂物证的检验鉴定领域以得到更加详细和准确的数据;将化学计量学方法应用到燃烧残留物谱图结果的分析中、研究助燃剂检验鉴定分析领域的人工智能识别技术,是未来进一步提高鉴定结果准确性和科学性的有效途径。
